# A quantum computing approach for minimum loss problems in electrical distribution networks

**DOI:** 10.1038/s41598-023-37293-9

**Published:** 2023-07-04

**Authors:** Filipe F. C. Silva, Pedro M. S. Carvalho, Luís A. F. M. Ferreira

**Affiliations:** 1 INESC-ID, Sustainable Power Systems Group, 1049-001 Lisbon, Portugal; 2 Physics of Information and Quantum Technologies Group, 1049-001 Lisbon, Portugal; 3 Instituto Superior Técnico, University of Lisbon, 1049-001 Lisbon, Portugal

**Keywords:** Electrical and electronic engineering, Energy grids and networks, Power distribution

## Abstract

This paper presents an application of a novel quadratic unconstrained binary optimization (QUBO) formulation to the minimum loss problem in distribution networks. The proposed QUBO formulation was conceived to be employed in quantum annealing—a quantum computing paradigm useful for solving combinatorial optimization problems. Quantum annealing is expected to provide better and/or faster solutions to optimization problems when compared to the ones provided by classical computers. With the problem at stake, better solutions result in lower energy losses, and faster solutions contribute to the same outcome given the future need for frequent reconfiguration of distribution networks to accommodate highly volatile demand, as anticipated by recent low-carbon solutions. The paper presents the results obtained through a hybrid quantum-classical solver for a standard 33-node test network and compares them with the ones obtained from classical solvers. Our main conclusion is that quantum annealing has potential to show advantage in the near future in terms of solution quality and time-to-solution, as quantum annealers and hybrid solvers continue to improve their performance.

## Introduction

Given the paradigm shift towards a reduced carbon footprint, electrical distribution networks are facing the challenge of handling increased levels of net load volatility introduced by the integration of low-carbon technologies such as distributed generation and electric vehicle charging while operating with minimum energy losses. Therefore, a network must quickly adapt itself through reconfiguration to its ever changing net load profile (actual and forecast) in order to be energy efficient^1^. In the reconfiguration process, the new network configuration is the optimal solution of the minimum loss problem for the new net load profile. To achieve the best possible network efficiency in terms of energy losses, it is crucial that an optimal or near-optimal solution of the minimum loss problem is possible to be found in the shortest amount of time, in order to be able to change the network configuration after the arrival of any update to the known net load profile.

Literature is rich in optimization approaches to the reconfiguration problem. The first report of such a problem for losses minimization dates back to 1975. Since then, hundreds of works have addressed different versions of the problem, tackling different objectives and constraints with a variety of heuristic solution approaches, such as using genetic algorithms^2,3^, particle swarm optimization^4^ and bacteria foraging optimization^5^. Also promising are the very recent methods for network reconfiguration based in machine learning^6–8^. A comprehensive survey of reconfiguration problems and solution approaches can be found in the literature^9^.

Quantum annealing^10^ is a quantum computing technology specially useful to solve combinatorial optimization problems like the minimum loss problem with the perspective of achieving better and/or faster solutions than the ones obtained with classical methods. In order to be handled by quantum annealing, the problem must be formulated as a quadratic unconstrained binary optimization (QUBO) model. There are not in the current literature any known methods for the reconfiguration problem which could be used in quantum annealing.

In our previous work^11^, we proposed a novel QUBO formulation for the minimum loss problem, paving the way to the application of quantum annealing to this kind of problems. The present paper contributes by demonstrating the feasibility of the quantum annealing solutions to those problems and by comparing the solutions quality and time against those returned by classical solvers for the QUBO model. The comparisons are carried out for the well known standard 33-node electrical distribution network^12^.

This paper is organized as follows. Quantum annealing is introduced next, ending this “Introduction” section. In the “Methods” section, our approach is described in more detail—the optimization problem is mathematically formulated and the building process of the QUBO model is introduced. The “Results” section presents the numerical results obtained for the solution of the QUBO model using several solvers. Finally, the “Conclusion” section summarizes our main results and describes the directions for our future work.

### Quantum annealing

Quantum annealing is one of the two main paradigms of quantum computing (the other being the gate model or circuit model). It can be employed to find the solution of discrete optimization problems. Compared with classical computers, quantum computing has the potential to provide an exponential speedup on problem solving, depending on the type of problem being solved. This speedup lies on the unique phenomena of quantum superposition and quantum entanglement which enables the computation parallelization with an exponential scaling with the quantum hardware size (i.e., the number of qubits—quantum bits) as opposed to the linear scaling with the number of cores of classical computing.

While the quantum gate model is able to handle more general computational problems (e.g., Shor’s algorithm for integer factorization^13^), quantum annealing is better suited to optimization problems like the one we are addressing. Although the gate model can deal with optimization problems through the quantum approximate optimization algorithm (QAOA)^14^, it suffers from the need to add quantum error correction to overcome qubit noise from nowadays Noisy Intermediate Scale Quantum (NISQ) implementations of gate-based quantum computers^15^. This error correction means that several physical qubits must be grouped together to represent a single logical qubit, thus limiting the number of logical qubits available to represent the problem variables. Also, these gate model implementations fall behind current annealers in terms of number of qubits and number of qubit couplings—the largest annealer currently available (from D-Wave) has more than 5600 qubits^16^ while the largest gate model quantum computer to date (from IBM) has 433 qubits^17^.

The energy of a quantum system is a function of the quantum state of this system. This function is called the Hamiltonian. The ground state of the system is defined as the state with the minimum possible energy as given by its instantaneous Hamiltonian. Thus, the ground state depends on that Hamiltonian. If a quantum system is in its ground state and if the Hamiltonian starts to be changed smoothly and slowly enough, then the quantum state of the system also changes such that it remains in the ground state of the changing Hamiltonian. This process is called an adiabatic evolution.

The adiabatic quantum computing theory^18^ shows how adiabatic evolution can be used to solve minimization problems, as follows. The quantum system starts with a simple Hamiltonian having a ground state that is easy to construct. Then, the Hamiltonian is adiabatically changed by the control system such that, at the end of the evolution, the Hamiltonian is a physical representation of the minimization problem cost function. This representation is such that the quantum system state and energy correspond to a problem solution and its cost, respectively. Given that the system evolved adiabatically, it will be in the ground state of the final Hamiltonian and thus representing the problem solution with the minimum energy, i.e., the optimal solution. The final quantum state is then measured and returned as a binary string representing the problem solution.

Quantum annealing is a real-world approximation to the theoretical adiabatic quantum computing. The undesirable thermal interactions between the quantum system and the surrounding environment make the quantum evolution deviate from the ideal case, resulting in a possible degradation of the solution quality. Other factors such as the accuracy of the physical implementation of the Hamiltonian representing the optimization problem may also affect the solution. These non-idealities, together with the probabilistic nature of quantum mechanics, make the quantum annealer a statistical sampler rather than a deterministic solver. The solution samples returned by the quantum annealer follow approximately a Boltzmann distribution, thus assigning a higher probability to solutions with lower cost. An optimal or near-optimal solution can be found with a given probability on a certain number of solution samples returned from the quantum annealer. This number depends on problem size and structure and it is usually found empirically since the exact solution distribution cannot be easily determined for a given problem.

The quantum annealer is able to implement Hamiltonians for minimization problems formulated in the QUBO model^19^. This model implements a pseudo-Boolean function^20^ representing the problem cost as a function of a binary string (a problem solution).

## Methods

### Problem description

Given the network connectivity graph $G=(V,E)$, the minimum loss problem consists of finding the spanning tree of *G* which minimizes the total ohmic losses on the network links, assuming that any link in *E* can be individually disconnected. More formally, the optimal spanning tree $T^*=(V,E^*)$, where $E^* \subsetneq E$, is given by

1 $$\begin{aligned} T^* = \operatorname*{arg\,min}_{T \in {\textrm{{ST}}}(G)} \sum _{(u,v) \in E(T)} R_{uv}\left| I_{uv}(T)\right| ^2 \end{aligned}$$

where $\textrm{ST}(G)$ is the set of all spanning trees of *G*, *E*(*T*) is the set of all links on *T*, $R_{uv}$ is the resistance on link (*u*, *v*) and $I_{uv}(T)$ is the complex phasor of the current on the same link with tree *T*. Since the network model defines no transversal link susceptance, the link current is simply the sum of the nodal load currents downward of the link, as given by

2 $$\begin{aligned} I_{uv}(T) = \sum _{n \in D_{uv}(T,v_0)} I^L_n(T) \end{aligned}$$

where $D_{uv}(T,v_0)$ is the set of all nodes downward of link (*u*, *v*) across *T*, i.e., the nodes with a path to the root node $v_0 \in V$ (the substation) along *T* which includes the link (*u*, *v*), and $I^L_n(T)$ is the complex phasor of the current drawn in node *n*’s load. The set $D_{uv}(T,v_0)$ includes one of the nodes *u* or *v* depending on *T*.

The network model used in this work defines the loads as PQ (i.e., constant active and reactive power)^12^, which is the typical case in power systems modeling. The nodal current $I^L_n(T)$ is given by

3 $$\begin{aligned} I^L_n(T) = \frac{\overline{S_n}}{\overline{V_n(T)}} \end{aligned}$$

where $S_n$ is the complex power consumed by the load (given as a problem input), $V_n(T)$ is the complex phasor of the nodal voltage for configuration tree *T* and ${\overline{c}}$ is the complex conjugate of *c*. The nodal voltage $V_n(T)$ is calculated from the voltage drop through the link connecting *n* to its parent node $p \equiv p_n(T)$, as given by

4 $$\begin{aligned} V_n(T) = V_p(T) - Z_{pn} I_{pn}(T) \end{aligned}$$

where $Z_{pn}$ is the complex impedance of link (*p*, *n*). The voltage of the substation ($V_{v_0}$) is fixed and typically equal to the network nominal voltage, i.e., 1 pu (per-unit). Equations (2), (3) and (4) define the radial power-flow for a network configuration *T*. This calculation must be performed iteratively since no closed-form expression from the power-flow solution can be derived.

#### PQ-load model

Given that $I_{uv}(T)$ cannot be expressed in closed form for PQ loads, the same applies to the problem objective function given in (1). In our previous work^11^, we proposed a QUBO formulation for this problem which models the network loads as constant current instead of constant power, which is equivalent to assume in (3) that $V_n(T) = 1$ pu (with a null angle) for all loads. This step was necessary in order to express the objective function in a closed form suitable to a QUBO formulation.

In the present work, we are proposing an iterative method to solve the original PQ-load problem using our QUBO formulation for constant-current loads. This method can be described in the following steps:

1. Build the QUBO model assuming constant-current loads, i.e., assume $V_n(T) = 1$ pu for all loads in (3).
2. Solve the QUBO model, extract the optimal configuration $T^*$ from the solution and add it to the list of visited configurations.
3. Calculate the voltage profile for the optimal configuration ($V_n(T^*)$ for all loads) using a classical power-flow method for PQ loads.
4. Update the QUBO model with the constant-current loads adjusted for the voltage profile obtained in previous step, i.e., recompute (3) using the values of $V_n(T^*)$ in the place of $V_n(T)$ for all loads.
5. Solve the updated QUBO model and extract the new optimal configuration $T^*$ from the solution.
6. If new $T^*$ is not in the list of visited configurations, add it to the list and go to Step 3.
7. Compute the power losses for all configurations in the list of visited configurations using a classical power-flow method for PQ loads and return the configuration with minimal losses.

This paper is mainly focused in the constant-current load model problem (i.e., Steps 1 and 2 above). In the end of “Results” section, the application of our proposed approach for PQ loads is presented for several standard distribution network models.

#### Voltage and current constraints

Although the proposed formulation does not include constraints related to voltage and current limits, these constraints tend to be fulfilled in a configuration optimized for minimal losses. Considering that constraint violations in the optimal configuration are possible but infrequent, penalty terms could iteratively modify the QUBO model to prevent violations from appearing. The evolution of the present work will include this approach to handle constraint violations in the QUBO model.

### The proposed formulation

The formulation we developed consists of creating a QUBO model representing a minimum loss problem for any given network topology, electrical parameters and net loads. The optimal solution of this QUBO problem contains the operational spanning tree which minimizes the QUBO cost function, i.e., the network energy losses in the constant-current load model.

**Figure 1:**
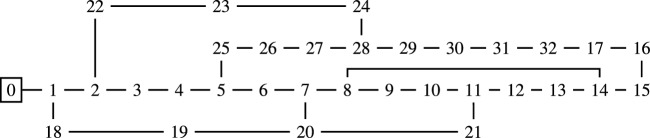
The 33-node test network; the substation is node 0.

Our formulation is described in detail from a mathematical point of view in our previous work^11^. The present paper is focused on the numerical results of the solutions obtained through several solvers for the minimum loss problem with our formulation applied to a standard 33-node test network. This network model^12^, represented in Fig. 1, is widely used to benchmark network optimization algorithms^9^. In this work we used the network model data from the Matpower software package^21^.

#### QUBO model

A QUBO model formulates a pseudo-Boolean cost function $f:{\mathbb {B}}^n \rightarrow {\mathbb {R}}$ as a quadratic polynomial over *n* binary variables $x_i\in {\mathbb {B}}$, where $i=1 \dots n$ and ${\mathbb {B}}=\{0,1\}$:

5 $$\begin{aligned} f(x)=\sum _{i=1}^n a_i x_i + \sum _{i<j}b_{ij} x_i x_j + c \end{aligned}$$

where $a_i$ and $b_{ij}$ are the real-valued linear and quadratic coefficients, respectively, and *c* is a constant term. Solving a QUBO problem consists of finding the binary string $x^*$ which minimizes *f*:

6 $$\begin{aligned} x^* = \operatorname*{arg\,min}_{x \in {\mathbb {B}}^n} f(x). \end{aligned}$$

While the constant term *c* has no influence on $x^*$, it’s inclusion on *f* makes this function more general in order to provide meaningful cost values for the optimization problem at stake.

For our optimization problem, the solution $x^*$ contains the model variables that we introduced in our previous work^11^. Thus, each of these variables is assigned to a given variable index *i* in (5). Each model variable is either a decision variable or an auxiliary variable. The decision variables represent the solution—in our problem they define which network links are in the edge set $E^*$ of the optimal spanning tree $T^*$. The auxiliary variables, whose values depend on the decision variable values, are needed to implement the problem constraints and objective function (i.e., the energy losses function) with the binary quadratic terms of the QUBO model. For our test network, the QUBO model has 47 decision variables (the *e* and *p* variables in our previous work^11^) and 1027 auxiliary variables, for a total of 1074 binary variables.

Since the QUBO formulation is, by definition, unconstrained, the problem constraints must be added to the cost function as penalty terms. These constraints include the topology constraints needed to restrict the solution space to valid spanning trees. Any solution which would violate some constraint has a penalty cost higher than the objective function, such that the solution is avoided in the total cost minimization even if there would be some possible benefit on the objective function. An extreme example of such case is a solution representing a completely disconnected network with null energy losses. The minimum penalty cost value per constraint violation is defined as 2.0. This is the default value of the tool we used to convert the constraint satisfaction problem into a QUBO model—the stitch function from the dwavebinarycsp Python package of the D-Wave Ocean SDK^22^.

Before being added to the total cost function of the QUBO model, the energy losses function is scaled by a constant factor such that the scaled losses value expected for the optimal solution (or for the best known valid solution) lies below the minimum penalty cost of 2.0. Nevertheless, the scaling factor cannot be too low, otherwise the model may be solved with some loss of numerical precision, yielding a sub-optimal solution. This issue is specially relevant for quantum annealers since there are limits on the range and precision of the physical implementation of the QUBO coefficients $a_i$ and $b_{ij}$^23^. We chose as the network starting configuration, i.e., the configuration which the solvers’ solutions will be compared with in terms of losses reduction, the valid and non-optimal network configuration with all five tie lines open—(7,20), (8,14), (11,21), (17,32) and (24,28). This configuration has energy losses of 165.4 kW. Thus, if we define the scaling factor as 0.01 for the energy losses expressed in kW, this configuration has a QUBO cost value of 1.654, below the minimum penalty cost of 2.0. Given that, for this network, the QUBO value of any configuration better than the starting configuration is still below this threshold, we fixed the scaling factor for this network as 0.01.

**Figure 2:**
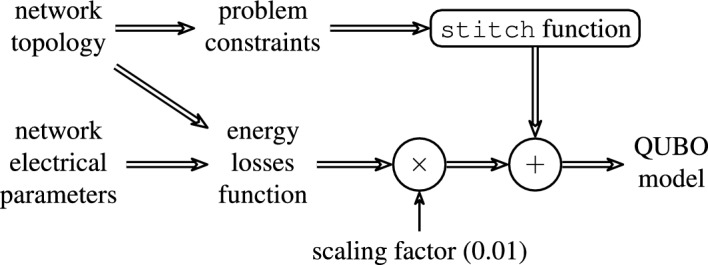
The building process of the QUBO model.

Figure 2 summarizes the process of building the QUBO model.

## Results

In order to find the optimal solution for a QUBO model, several solvers can be used. Three approaches were studied with our model—classical solvers, quantum solver (quantum annealing) and hybrid quantum-classical solver. To benchmark the solvers results, the optimal solution for the constant-current load model was found by exhaustive search over the complete 50,571 spanning trees space of the 33-node test network. This solution is represented by the set of open links {(6,7), (8,9), (13,14), (31,32), (24,28)} with energy losses of 116.4 kW, thus corresponding to a QUBO cost value of 1.164.

### Classical solvers

Table 1 summarizes the results obtained from classical state-of-the-art QUBO and mixed integer quadratic programming (MIQP) solvers. Given the stochastic nature of these solvers, the results vary between distinct runs but they keep the same order of magnitude for both the incumbent solution value at a given moment of the run time, and for the time-to-solution (TTS) if an optimal solution is found. The FICO Xpress, CPLEX and SCIP solvers were the only ones to find the optimal solution, being FICO Xpress the fastest solver with a TTS of 48 s. The NEOS Server platform^24–26^ limits each optimization job to four threads, while the Amazon EC2 t2.micro instance^27^ runs with a single virtual core. It would be expected that the MIQP solvers would be able to find the optimal solution within a smaller TTS with a mixed integer constrained formulation for this optimization problem instead of a QUBO formulation. Nevertheless, the focus of this paper is the comparison of the results from several solvers with the same QUBO formulation.

**Table 1:** Solution results obtained from classical solvers.

Solver	Platform	Solution value	Run time ([h:]mm:ss)
FICO Xpress^28^	NEOS Server	1.984	00:30
		1.164^a^	00:48
CPLEX^29^	NEOS Server	2.630	05:00
		1.164^a^	05:16
SCIP^30^	NEOS Server	4.164	05:00
		1.164^a^	22:46
AlphaQUBO^b^^31^	Amazon EC2 t2.micro	4.203	00:30
		4.179	01:00
		2.340	05:00
		2.321	15:00
		2.629	30:00
MQLib^c^^32^	Amazon EC2 t2.micro	4.556	00:35
		2.364	04:00...15:00^d^
Gurobi^33^	NEOS Server	6.151	00:30
		4.171	1:00:00
		2.958	2:30:00...8:00:00^d^

^a^Optimal solution value.

^b^The results of AlphaQUBO are from distinct runs with different time limits. The solution cost value for 30 min run is higher than the one for 15 min given the stochastic nature of the solver while it cannot improve the solution any further within the given time frame. The results of the other solvers are from a single run on each solver.

^c^The results of MQLib are from the GLOVER2010 heuristic^34^, which is the best performing heuristic from the 39 MQLib heuristics tested with our model.

^d^The dots represent a time interval were the incumbent solution value was stationary.

#### Solving with an initial feasible solution

In order to improve the solution and/or the TTS, an initial feasible solution was given to the solvers being able to accept it: CPLEX and Gurobi. This solution corresponds to the valid and non-optimal network configuration with all five tie lines open which, as previously mentioned, has a QUBO cost value of 1.654. The value for the 1074 solution variables was directly assigned from the network configuration. Table 2 summarizes the results obtained from these two solvers. CPLEX was able to find the optimal solution in about half of the time it took to find the same solution without an initial solution. Gurobi was still not able to find the optimal solution, showing only a small improvement after about 3 h of run time.

**Table 2:** Solution results obtained from classical solvers with an initial feasible solution.

Solver	Platform	Solution value	Run time ([h:]mm:ss)
CPLEX	NEOS Server	1.654	00:00...02:37^b^
		1.164^a^	02:50
Gurobi	NEOS Server	1.654	0:00:00...2:39:20^b^
		1.412	3:00:00...8:00:00^b^

^a^Optimal solution value.

^b^The dots represent a time interval were the incumbent solution value was stationary.

### Quantum annealing

We had access to the D-Wave Advantage quantum annealer^16^. This system is currently the largest quantum annealer with more than 5600 qubits and 40,000 qubit couplings although it has a limited connectivity between the qubits, with a maximum of 15 couplings per qubit. Each qubit represents a binary variable $x_i$ from a physical QUBO model and each qubit coupling implements a qubit product term $x_i x_j$ from the same model. Both the qubits and the qubit couplings are programmed with their QUBO coefficients $a_i$ and $b_{ij}$, respectively.

Given the limited connectivity of the physical qubit couplings, the QUBO model variables of an optimization problem may not have a one-to-one correspondence with the annealer qubits. In this case, the graph representing the QUBO model (where the nodes are the model variables and the edges are the product terms) must be embedded in the annealer connectivity graph—the Pegasus graph^35^. Since the former graph must be a minor of the latter graph, this procedure is a minor embedding where each model variable corresponds to one or more annealer qubits^36^.

Although our model’s 1074 variables and 10,166 product terms are well below the annealer limits, no embedding is currently possible given the high connectivity of most of our model variables. A future quantum annealer with more qubits and/or qubit couplings will eventually make the minor embedding possible for our QUBO model.

### Hybrid solver

**Figure 3:**
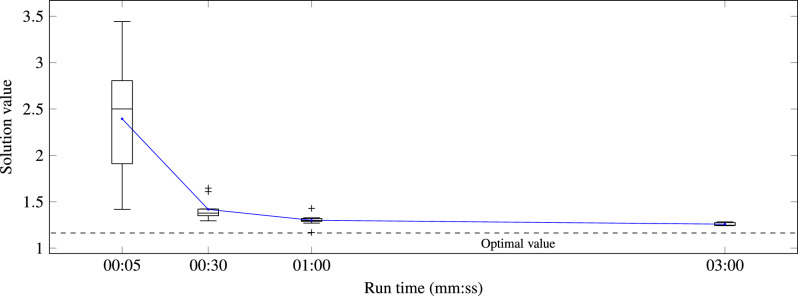
Solution results obtained from the D-Wave hybrid solver. The box plots and the line represent the statistics and the average, respectively, of the solution values for the each of the chosen run time limits.

The D-Wave Hybrid Solver Service^37^ aims to overcome the quantum annealer limitations on size and connectivity while still taking advantage of the acceleration provided by quantum annealing. No minor embedding is needed for the QUBO model supplied to this solver. This service runs several classical heuristics in parallel. Each of these heuristics makes queries to the quantum annealer with subproblems which fit on the annealer. Since this solver is proprietary, we do not have access to its implementation details. However, from the timing information returned in the solver solutions we conclude that about 1/60 of the hybrid run time is allocated to quantum annealing runs. Figure 3 and Table 3 summarize the results obtained from this solver. The solutions were obtained by defining a time limit to each run. After reaching this limit, the solver returns the best solution found in that period. Given the stochastic nature of this solver, several runs were made for each chosen run time limit in order to obtain statistic information about the solution values.

**Table 3:** Solution results obtained from the D-Wave hybrid solver.

Run tim (mm:ss)	Solution value			Feasibility ratio (%)	Runs
	Min	Average	Max		
00:05	1.418	2.394	3.442	40	10
00:30	1.295	1.418	1.647	100	10
01:00	1.169	1.300	1.428	100	10
03:00	1.242	1.258	1.283	100	5

All solutions with a value below 2 are feasible since a constraint violation would add a cost of at least 2. Still, the feasibility of each solution was checked directly against the problem constraints. All solutions returned for a run time of 30 s or longer and 40% of the 5 s solutions were shown to be feasible.

As expected, the solution values generally decrease as the run time increases. The standard deviation for each time limit shows the same evolution. It worth mentioning that the value of the best solution found for a run time of 1 min has a difference to the optimal value of just 0.00486 (i.e., a difference of 486 W in total power losses) and with a single difference from the optimal set of open links shown in the beginning of the Results section. This solution is better than all solutions found for the longer run time of 3 min. This exceptional situation is justified by the stochastic nature of this solver and by the fewer runs made for the longer run time.

Considering the already mentioned non-optimal network configuration with the tie lines open (with a QUBO cost value of 1.654) as the starting point, the solutions returned from the hybrid solver yield on average 48% of the maximum possible reduction in losses for a run time of 30 s. The best solutions returned for a run time of 5 s and for a run time of 30 s showed a losses reductions of 48% and 73%, respectively.

All the solutions returned by the hybrid solver showed no improvement after being post-processed with a classical greedy steepest descent solver provided by D-Wave^38^. The greedy solver operates by flipping one variable at a time to try to improve the solution. Since no improvement was observed, one can conclude that solutions returned by the hybrid solver are locally optimal for a neighborhood defined as a Hamming distance of one.

### PQ-load model

The results previously presented were obtained for the constant-current load model. In order to solve the original PQ-load problem, the iterative method we proposed in Methods section was applied not only to the 33-node network already employed, but also to 70-node^39^ and 118-node^40^ test networks. As with the 33-node network, the source of the model data for the larger networks was also the Matpower software package^21^.

Since the focus of this subsection is to illustrate the application of the proposed PQ-load method for several test networks, we are not concerned here on how the QUBO model is solved within the proposed method. Thus, the QUBO model was replaced by an equivalent MIQP model more suitable to classical solvers which provide a guarantee of optimality. This MIQP model represents the same optimization problem as the QUBO model. The main difference is the representation of link currents as continuous variables, enabling a simple formulation of the network losses as a quadratic function of these variables. This observation does not remove the merits of the QUBO model since this is the only model that a quantum annealer can directly accept.

Table 4 summarizes the results obtained for the validation of the proposed PQ-load method using the Gurobi solver. This table shows that the method converged in the first iteration for all tested networks, i.e., the optimal configuration for PQ-load model (Step 5 of the method) is the same as the optimal configuration for the constant-current load model (Step 2) in the first iteration. Although there is no guarantee that the method converges at the first iteration for any possible network, we strongly believe that the method converges at most within a small number of iterations.

**Table 4:** Results obtained from the proposed method for PQ-load model.

Network size (nodes)	Iterations	Optimal losses (kW)	
		Const. I	PQ-load
33	1	116.4	127.7
70	1	263.5	301.1
118	1	789.0	865.0

## Conclusion

This paper proposed an application of a QUBO formulation of the minimum loss problem in distribution networks to a quantum-classical hybrid solver. The results of this application were compared against state-of-the-art classical solvers for the standard 33-node test network. The D-Wave hybrid solver found good solutions within a reasonable run time. Although the exact optimal was never found, a solution very close to the optimal was found with a run time of 1 min. The worst solution returned by this solver for a run time of 30 s was still better than any solution returned by other solvers in the same run time (including the classical solvers with an initial feasible solution). Several classical solvers were able to find the optimal solution, being the FICO Xpress solver the fastest one with a time-to-solution of 48 s.

Since our QUBO formulation applies to constant-current load models only, we proposed an iterative method to optimize PQ-load models using the QUBO optimization as an inner step. This method showed an immediate convergence with three standard network models between 33 and 118 nodes.

Other methods may currently provide better or faster solutions to the minimum loss problem. Nevertheless, as quantum annealers and hybrid solvers will continue to improve their performance, we expect that the proposed QUBO formulation will enable the practical use of quantum annealing or quantum-classical solvers for handling reconfiguration problems on real-world electrical networks with advantage over classical solvers in terms of solution quality and time-to-solution.

The evolution of the present work will include the addition of voltage and current constraints to the QUBO formulation. This evolution will also target a new QUBO formulation with a better scaling of the model size with respect to the network dimension.

## Data Availability

The network models data used in this study is included in the Matpower software package (https://matpower.org/). The QUBO and MIQP models generated during this study and the solution data obtained from the solvers are available from the corresponding author upon reasonable request.
